# Tuberculosis, from Anatomical, Etiological, and Preventive Standpoints, with Especial Reference to Phthisis Pulmonum*Read before the Mass. Veterinary Association at Boston, 1885.

**Published:** 1886-01

**Authors:** Frank S. Billings

**Affiliations:** Late Pathologist to the New York Polyclinic School of Medicine


### ART. II.—TUBERCULOSIS FROM ANATOMICAL, ETIOLOGICAL, AND PREVENTIVE STANDPOINTS, WITH ESPECIAL REFERENCE TO PHTHISIS PULMONUM.*

BY FRANK S. BILLINGS, V.S.,

*Late Pathologist to the New York Polyclinic School of Medicine.*

### THE HISTORICAL DEVELOPMENT OF THE VIEWS WITH REFERENCE TO TUBERCULOSIS.

Before entering upon the discussion of this very interesting question, the salient points of which we shall only be able to touch upon, it is necessary that we come to an understanding as to what is at present accepted as the meaning of the words tubercle and tuberculosis. Of necessity the two need have no connection with one another. That tubercles can develop in the lungs of an organism without being followed by phthisis is well known. According to the modern school of bacterio-pathology it is asserted that all forms of pulmonary phthisis are tuberculous. The word "tubercle" is specific with reference to the limitations of size and form only. The word "tuberculosis" is limited to an etiological conception.

Tubercles may be met with at times in almost every organ, but more especially in the lungs of almost every form of animal life. What is at present termed genuine tuberculosis is limited to the anthropomorphic and bovine species in the first place, and secondly, to swine. It may be transmitted however, to almost every form of warm-blooded life. The tubercles so frequently met with in the organs of a large number of our domestic, as well as wild animals, are almost invariably due to the irritative action of some animal parasite, or occasionally to some inanimate foreign material. A very marked development of tubercles is frequently seen in actinomycosis. It is, therefore, evident we should not use the word tubercle without a qualifying adjective, except in a morphological sense. Nodulus and tuberculum should be identical in meaning now as formerly.

* Read before the Mass. Veterinary Association at Boston, 1885.

They have reference to form, and were only used in that sense by the fathers in medicine, the same as they used the words polypus and fungus. It was but natural that early observers should find their attention chiefly attracted by the morphological appearances of neoplastic productions. The fathers, however, used their powers according to the light that was given them, and did not limit their descriptions to morphology alone, but also gave consideration to the density, color, contents, etc,, of such products. So far as the eye could see, or the hand feel, they described quite correctly; hence we frequently read of a "Tuberculum scirrhosum," or "durum," or "scrophulosum," as well as of polypi or fungi of the same nature.

Neither form, consistency, nor color are, however, characteristics upon which we can place any great value with reference to the elementary structure of neoplastic products. From the etiological point of view such macroscopical phenomena are valueless. Hippocrates and the earlier Greek writers frequently used the word "Phymata" to characterize the same thing for which their Latin successors used "Tubercula;" but neither of them applied either word to the noduli to which modern usage applies the word "tubercle." On the contrary, these words were used indiscriminately for almost every variety of circumscribed product, abscesses, bubos, hypertrophied glands, tumors, etc. Sylvius, 1680, appears to have been the first observer that described anything which bears any morphological resemblance to the existing ideas of tubercle. Things remained "*in status quo ante*" until the birth of the analytical school of medicine, through the agency of Bichat, in the latter part of the last century in France. The advancing minds of this school soon found themselves in a hot fight as to what was tubercle, what tuberculosis, and what scrofulosis. Bayle and Baillie were among the first to enter the pathological arena, but the Bayard among these medical knights was undoubtedly the father of pulmonary pathology, the genial and immortal Laennec.

Laennec by no means differentiated the tubercle of phthisis from other morphologically or macroscopically similar products; in fact, he seems to have almost entirely ignored the existence of the tubercle, *per se*, in phthisical processes, and to have given his attention to the terminal result in such processes, viz., caseation. In his writings we find tuberculization equivalent to caseation—tubercular infiltration and scrofulosis all treated as one and the same thing. The tubercle owed its origin to a scrofulous diathesis, without which it could not develop. At this time we find the writings full of discussions as to what was "Scrofel-stoff" or "Tuberkel-stoff," and whether they were identical materials or not. The views of this period, somewhat modified by time and experience to be sure, are very well expressed by Sir Robert Carswell in his atlas. He says:

"The term Tubercle is employed to designate a peculiar morbid product which pathologists describe as occurring in various organs in the form of a small round body, said by some to consist at first of a firm, gray, somewhat transparent substance which afterwards becomes opaque and of a dull yellow color, and may then be broken down between the fingers like a morsel of cheese. These characters are said to represent what is called the first period, or true tubercle. At some subsequent, but indefinite period, the crude tubercle loses its primitive consistence in virtue, it is believed, of certain inherent properties by means of which it is converted into a liquid mass, of the consistency of cream." It is further stated that, " this process, which constitutes the period of softening, is perceived always to take place in the centre of the crude tubercle, and proceeds from thence to the circumference."

Andral, in particular describes the tubercle at its origin, as a pale yellow opaque small round body of various degrees of consistency, and in which no trace of organization can be detected. He denies that the gray semitransparent corpuscle, or granulation, as described by Laennec and since by Louis, constitutes the primitive state of the tubercle, or that the process of softening takes place invariably in the centre of this substance. The latter changes he ascribes to the admixture of pus, secreted by the tissues subjected to the stimulus of the tubercle, as a foreign body, and not to any change originating in the tubercle itself. He conceives a correct definition of tubercle, or rather of tuberculous matter, which constitutes the essential anatomical character of those diseases to which the term "tubercular" is now so exclusively credited, to be as follows: Tuberculous matter is a pale yellow, or yellowish gray opaque unorganized substance; the form, consistence and composition of which vary with the nature of the part in which it is formed and the period at which it is examined. The prevailing opinion among pathologists is, that the seat of tuberculous matter is the cellular tissue of organs; that it may however be formed on secreting surfaces as in the numerous follicles of the intestines; perhaps in the air cells and bronchi; the surface of the pleura, and likewise in false membranes, or in other accidental new products, and in the blood itself, *i. e.*, may be an Exudate.

Schonlein wrote, 1832 that "the tubercle family is related to the scrofulous, but by no means identical with the same. Tuberculosis appears only in the cellular tissue, and especially in that of secreting organs. The tubercle is to be looked upon as something foreign; the organ therefore shows a tendency to throw it off. This tendency is shown in various ways according to the functional variation of the organs: *i. e.*, in tuberculosis of the lungs by means of coughing, which is not to be looked upon as a symptom of disease, but rather as an indication of the curative attempts of nature. In tuberculosis of the stomach or liver nature shows her efforts in this direction by emesis; and when the nervous system is complicated, especially in tuberculosis of the brain, by convulsions. Anatomically considered, we observe in the tubercle a nucleus and an enclosing membrane. The latter is either formed from the compressed cellular tissue of the organ in which the neoplasm is situated, or it is a newly developed membrane taking its origin in the elements of the same. Sometimes this capsule consists of several layers; an external or vascular, and an internal, or serous membrane; a construction which reminds us of the Corion and Amnion.

"The nucleus undergoes a certain cyclus of change, which we call tubercular metamorphosis. In the beginning it is more or less transparent and is composed of a sort of gelatinous mass. Later on it becomes clouded, under conditions which are to be compared to yolk development in the ovum of lower animals. This cloudiness gradually extends in a radial direction towards the peripheries until the entire tubercle loses its transparency and is transformed into a mass of fatty detritus. According to our present knowledge every tubercle is at first a cyst, 'Blase,' filled with gelatinous fluid and of a roundish form."

Henle, 1847, speaks of the "tubercle as an organized formation. These bodies which are sometimes looked upon as a raw product, consisting of a blastema incapable of further development, and at others as malignant products having peculiar vital characteristics which justifies their classification with parasitic neoplasms, are neither the one or the other, but are bloodless, necrobiotic lobuli which remain in connection with the healthy parts. They are filled with epithelial elements, or with pus cells, granular conglomerates and granular cells that have suffered disturbances in their regular course of development, and have been conserved in a desiccated condition with the substance in which they are enclosed. We place a certain value upon the form, from the ontological standpoint, when the tubercle appears as a regular attribute, and at the same time as a distinguishing characteristic of a parasitic organism."

It is worthy of note that Bennett, 1842, mistook the fibrils forming the stroma of the tubercle for vegetable fungi, and described them as such.

Rokitansky was, in his earlier days, a literal follower of the Laennec school, and looked upon tubercle material as an exudate, but later (1855), describes the tubercle as "distinguished by its inclination to desiccation, with consecutive destruction of the tissues in which it is situated, on account of the high degree of development in its elements. The tubercular mass is a gray, semi-transparent material of a pearly color, or a yellow, opaque product. There is no doubt that this is a secondary stage of the originally gray-appearing elements. This metamorphosis is however not peculiar to the tubercle mass alone, but occurs also in other cellular and nucleoid products."

Burckmueller describes the tubercle "as a neoplasm, made up of lymph cells, which assume a nodular form. These noduli seldom attain the size of a millet seed; they are soft, of a grayish-yellow color, and consist of a densely accumulated mass of cells, without any intercellular substance; these cells are colorless and have a somewhat granulated plasma with divided nuclei; towards the peripheries of the nodulus we often find larger cells with several nuclei which resemble pus cells, as well as very large masses of protoplasma, with numerous nuclei (giant cells). The most common metamorphosis which the tubercle undergoes is caseation to which authors have previously given the name of 'Tuberculisation,' wherever met with. The tubercle is to be looked upon as a malignant neoplasm, because it has the ability to extend to juxtapositional tissues and to cause the ulcerative destruction of the same. The softened tubercular detritus often causes a species of infection which may lead to death by inflammatory processes in the lymphatics and lymph glands, and the accumulation of lymph cells (leucocytes) in the blood and metastasis."

It must be evident that it was largely through the influence of the Laennec school that the idea of the identity of scrofulosis and tuberculcosis obtained so great an authority and such a wide extension among pathologists. It remained for Virchow to show that caseation frequently occurred as the final result under conditions, and in connection with processes, that had neither an anatomical nor etiological connection with tuberculosis. He demonstrated that caseation was a deuteropathic, following upon a protopathic, or formative condition, and was, indeed, the gradual and incomplete death—Necrobiosis—of the cell-elements of a large variety of neoplastic processes. He taught that the genuine opalescent was the only true or vital tubercle, while the opaque, cheesy degenerated nodulus, was no more a tubercle but a dead mass. He drew the sharpest distinction between scrofulosis and tuberculosis, and insisted that either could occur entirely independent of the other, though the first might act as a predisposing cause to the second, or might owe its existence to constitutional conditions which, under other circumstances might lead to tuberculosis. He also differentiated very sharply between caseous bronchitic conditions, caseous pneumonia and tuberculosis, looking upon the tubercle as the result of the specific irritation of an unknown quantity (agent), upon the connective elements of the lungs. He asserted that, while a tubercle could develop from the connective septa into the lumen of an alveolus, and appear as a caseous plugging of of the air cell, still that it was not paranchymatous in its origin in a strict anatomical and genitive sense. He admitted that in the beginning tubercular conditions bore the strongest resemblance to those of caseous pneumonia, but asserted that miliary caseous broncho-pneumonia, in its first stage, could always be distinguished from miliary tubercular phthisis.

A point which must not be passed over was a "child of the period," the doctrine of specific tubercle cells or bodies; the "Tuberkel Körperschen" of Lebert. At the same time we may read detailed descriptions of all sorts of specific cells, such as those of glanders, cancer or syphilis. Such specific cells do not exist. The abnormal appearance, arrangement or position of cells is what constitutes anatomical or physiological specificity. All neoplastic cells are the derivatives from their normal, embryonal prototypes.

The idea of specific cells in tubercles again found expression in connection with the so-called "giant cells," to which we shall presently refer.

Virchow denied an inflammatory origin to the tubercle, as well as that it was an exudative product. This is about the condition in which Virchow left the tubercle question, and conforms to his present teachings from the anatomical standpoint.

I have previously alluded to the fact that the "giant cells" in tubercles were looked upon as pathognomonic objects. Virchow mentioned their occurrence in tubercles, but Langhans first studied and accurately described them so that they have frequently been spoken of as "Langhans'-cells." Their specificity is at present denied by most authorities, but the conditions of the question will be best shown by a few quotations.

Birch-Hirschfield says of them: "The endeavor to make the 'giant cells' a necessary element of the tubercle has been generally contradicted. The extensive multinuclea masses of the protoplasm, which we call 'giant cells,' occur under a great variety of conditions. Physiologically they appear as an element in bone-structure, and again where osteoid tissue is subject to resorption; in the uterine sinuses; in inflammatory products in experimental peritonitis; in the alveoli of the lungs in chronic pneumonia; in syphilitic inflammations and hyperplastic lymph-glands, and in certain tumors."

Herring, Ziegler, Perls, and others, all give similar testimony. Ziegler has seen them develop in connection with other cells between two thin pieces of glass, forming a capillary space when introduced into the sub-cutis.

Weigert* says: "They deserve the name of 'giant-cells,' not only on account of their size, but on account of their multinucleated condition. Among the varieties of giant-cells which are known to us, those of Langhans assume a peculiar position, because of the striking grooping of the nuclei which are not distributed promiscuously through the protoplasm of the cell, but have a decided tendency to accumulate towards its circumference, thus leaving the greater part of the cells free. Sometimes they form a continuous mantle, and at others a sort of necklace or belt, while at others they are collected at the poles of the cell. They are frequently oval instead of being round in form, while at other times they have a radial appearance. Although very frequently present in giant-cells, they are not to be considered as a constant, necessary, anatomical characteristic. It would be an error to look upon them as such."

It is a well-known fact that the specific bacilli of tuberculosis are present in considerable numbers in giant-cells, especially in the peripheries of the non-nucleated portion of the plasma, or in the nucleated part and between the nuclei. Weigert looks upon the bacilli, in this location, as playing an essential rôle in causing caseation of the plasma of the cell, and has noticed that, as this material becomes necrobiotic, the bacilli either die and disappear, or move to the still vital portions of the plasma. If by this he means us to understand, as he says, that the specific tubercle bacilli are the cause of cheesy necrobiosis of the cells in tubercular phthisis, then I must emphatically deny the hypothesis.

We have now arrived at a period which marks another great turning point in the history of tuberculosis. It may well be called the etiological-diagnostic or bacterial period.

Its founder is Robert Koch. Again, it will be best to let others tell us what are the existing views of this school.

Friedlander says: "Pulmonary phthisis should be classed among the forms of tuberculosis; the differentiation advocated by Virchow and Reinhardt between caseous pneumonia, case-

* Deutsche Med. Wochenschrift, Aug., 1885.

ous bronchitis, and genuine tuberculosis must still be adhered to, from the descriptive anatomical standpoint; but this point of view must be modified or dropped when we come to consider the histological conditions, more especially, however, when we take into account the etiological and practical points from which these conditions are also to be considered. The exact histological examination of the products in caseous pneumonia and bronchitis shows the same elementary composition with the so-called tubercle. In every phthisis, even in apparently non-tubercular forms, one can always find the characteristic, sub-miliary, non-vascular noduli with giant-cells, etc., which comprise the chief histological type of the fully developed tubercle."

He further says: "That the daily observation of the appearance of a tubercular pleuritis in the course of a caseous pneumonia, cannot any more be looked upon as the eruption of another disease, but far more probably as the extension of the processes which had previously existed in the lungs."

The discovery by Koch of the bacillus of tuberculosis has proven to the greatest certainty the unity of these processes, that phthisis and tuberculosis are due to the same etiological unit. Therefore, pulmonary phthisis must be looked upon, in most cases, as local tuberculosis. The presence of the specific bacilli of Koch in the sputum of an individual must be accepted as conclusive evidence that tuberculous processes are developing in the respiratory apparatus.

Continuing in the same line of argument, Orth says: "In order to avoid any misconception, I wish to emphasize the fact that, when we speak of inflammatory caseous processes ("Broncho-pneumonia caseosa") and those of tuberculosis, that the difference is only of an anatomical character, for, from the etiological point of view, for which their frequent combination is evidence, they are one, and all the effect of the action of the same irritant, the bacillus Kochii, which we are enabled to demonstrate, not only in the genuine tubercle, but also in genuine caseous pneumonia. If we name this irritant "tuberculous" and the disease and all diseased changes which it produces "tuberculous," then probably all caseous, broncho-pneumonias, etc., must, in this sense, be looked upon as "tuberculous," also. In order, however, to distinguish those affections which are primarily characterized by the development of tubercles, I would prefer to call the irritant scrofulous, and the disease scrofulosis, the anatomical conditions in general scrofulous, and only that tuberculosis which is distinguished by the primary development of tubercle."

To my mind these bacterio-pathologists are assuming altogether too much in their assertions with reference to the etiological connection of Koch's bacilli with all the processes that occur in the lungs in tubercular phthisis, but more especially caseous pneumonia. Admitted that they are the cause of a specific variety of tuberculosis, I am equally certain that the bacilli are not the direct cause of the cheesy degeneration in the neoplastic cells, either in tubercular phthisis or caseous pneumonia, or that they have anything directly to do with the necrobiotic destruction of the stroma of the lung, which terminates in the formation of cavities.

In "Bronchitis verminosa" of sheep, we find there is frequently a development of tubercles having many of the morpho-histological characteristics of the freshly developed bacillus tubercle. The animals die of marasmus (phthisis) but we do not find that caseous conglomerates or cavities develop in the lungs. These tubercles undergo cheesy degeneration and calcification; sometimes they form into fibroid noduli. This variation in the terminal results in the tubercle in sheep —the same is true of the tubercle in equine glanders—has nothing whatever to do with its cause, the strongylus, but is dependent upon the physiological condition of the lungs of the animals.

There is no evidence that the artificial tuberculosis induced in dogs and animals (having no natural or acquired predisposition to phthisical processes in the lungs) either by the aspiration of pure cultivations of Koch's bacilli, or by inoculations with the same, is ever followed by anæmic destructive processes which characterize tubercular or other forms of human phthisis. Such animals perish from a general marasmus due to disseminated tuberculosis, and not from phthisis pulmonum.

Weigert's theory that the bacilli are the cause of the caseous degeneration of the plasma of the giant-cells is utterly improbable. It is an attribute of these and other cellular products in phthisis, whether tuberculous or not, to undergo caseation. The plasma of such cells in such a condition being not only dead, but desiccated, no longer offers suitable conditions for the existence of the bacilli. Hence they leave such portions of the giant-cells and go to the still vital parts of the plasma.

This condition of the plasma may also have something to do with the peculiar arrangement of the nuclei as mentioned by Weigert. They cannot find the elements necessary to their vitality in a dead and dry plasma any more than the bacilli. That the presence of the bacilli in the cells may hasten caseous degeneration and desiccation of their plasma by entering into the struggle for existence, for both moisture and nutriment is apparent, but that they cause it, is quite another thing.

It would be fully as logical to assume that these bacilli were the cause of caseation in epithelial neoplasms, inspissated pus, retentive conditions, or syphilitic gummata, as in the lungs. The fact is, that caseation is the natural physiological direction inherent in certain cellular neoplastic products under certain inherent organic conditions, which they derive from their matrix, and neither the bacillus, or the tubercle *per se*, have anything whatever to do with it. Such cells are, like the organisms in which they develop, marked with death the moment they are born. That cases of caseous miliary broncho-pneumonia, in a disseminated form, can occur without necessarily terminating in either tuberculosis or phthisis pulmonum is so highly probable, that it can scarcely be questioned. The difficulty is to get autopsies in just such cases.

The constitutional weaknesses—phthisical or scrofulous diathesis in the lungs, which may primarily give nutriment to the bacilli in one case, may, in another, result in broncho-pneumonia caseosa, or the latter may so augment such conditions as to open the way for the attack of the bacilli. If tuberculosis follows or complicates such a disturbance, it is certain that an exposure to contagium has taken place. It is worthy of remark that not only the bacillus-tubercle (tuberculosis) but also all pseudo-varieties take their origin in the stroma and not in the paranchyma of organs. That the bacillus-tubercle is more prone to caseation than the pseudo varieties has already been noticed. The tissues in which they develop are weak and irritable, and their products must be of the same nature. In the first place, the irritation set up by the bacilli, in the intersticial tissue of the lungs leads to a local, circumscribed, disseminated nodular intersticial pneumonia, but on account of the non-resistant nature of the tissues, and from the fact that they form the septa and walls of the alveoli, the irritation at once extends to the same and is followed by an active proliferation of their endothelial cells, which bears no relation to the same in either a catarrhal or fibrinous pneumonia. In fact, so rapid does this proliferation go on, that the transudation of serum and the leucocytic migration which occurs in other pneumonias has no time to take place before the alveolus is densely packed with this cellular product. These cells have the same constitutional tendency to cheesy degeneration as those of the tubercle, though their matrix is different. This tendency to caseation and desiccation is increased by the cells themselves, from the pressure which such a putty like, immovable mass exerts upon the delicate reté of capillaries which embrace the alveoli. This net-work, being thus compressed between the solid mass filling the alveoli, becomes bloodless, while the non-diseased yet weak parts of the lung become hyperæmic through collateral fluxion, thus offering still more favorable conditions for the extended action of the bacilli, and the development of caseous-pneumonic infiltrations. The absolute anæmia thus produced by pressure of the caseous thrombi filling the alveoli must be the cause of the necrobiotic death of the pulmonary stoma in such parts which leads to the formation of cavities.

Thus it is evident that neither the bacilli nor the tubercle, nor even the caseous thrombus filling the alveoli, cause this destruction of the lung tissue through any materia peccans in the first place, though the ichorous material frequently resulting as large cavities are formed, may finally take its part in fulfilling the programme in this sad drama of life.

Summing up, then, tuberculosis is a disease characterized by the development of visible or submiliary noduli, having a grayish, somewhat opalescent appearance, and an apparently rounded form. These noduli are composed of very delicate fibrillary stroma and small epitheloid or round cells and, in general the protoplasmatic masses known as "giant cells;"

they are non-vascular. They are largely neoplastic productions, due to the action of a specific irritant upon the stroma of an organ, discovered by Koch, and otherwise known as the bacillus of tuberculosis. That which we have described is the true or living tubercle.

When it has become yellow and opaque, *i. e.*, caseous, it is no more vital, and is then much more difficult of differentiation from other neoplastic products that also undergo cheesy metamorphosis in the lungs. They are frequently so small, in the fresh invasion of densely paranchymatous organs, such as the liver, spleen and kidneys, that, when disseminated over the organ, it is impossible to see them without the aid of the microscope. The genuine tubercle possesses another attribute which distinguishes it more or less sharply from all pseudo varieties. While the latter generally appear as pretty clearly defined neoplasms, they are also isolated productions, even though they may present themselves to the microscopic eye as in apparently appositional relation to one another, still they do not have the faculty of extending and then forming a conglomerated mass; the true tubercle has the faculty (due to the bacilli) of invading adjoining tissues and thus extending indefinitely. Aside, however, from these characteristics, and aside from all previously employed methods of histological analysis, the true tubercle is always to be differentiated from all pseudo varieties by the demonstration of the presence of the specific bacillus.

The method for demonstrating these bacilli should be known to every physician, but its importance will warrant its re-statement. There are two which have been proved to be trustworthy, and have been generally adopted. The first is known by the names of those who perfected it, as the Ehrlich-Weigert method. A mixture known as analine water is prepared by adding 5 parts of analine oil to 100 parts of aq. dest. and shaking the two well up in a test tube. Then filter into a convenient, moderately flat and not too large glass dish. To this fluid we add about 5 parts of a saturated alcoholic solution of gentian, violet or fuchsin. To examine sputum one takes a little of the floculent sediment and spreads it out as thinly as possible upon a covering glass. After allowing it to dry in the air, the glass is to be grasped with a pair of forceps and passed three or four times through the flame of a Brunsen burner or spirit lamp, the material side to be held up. This process causes the albumen to coagulate and everything to adhere to the covering glass. One should always prepare at least six covering glasses with sputum from a single patient in this way for the first examination.

To show the constancy with which the bacilli may be found in the sputum from phthisical patients, it may be mentioned that Gaffky examined the sputa from 12 different persons, 982 times in all. He found the bacilli 938 times. The coloring mixture is then warmed until the vapor is seen to begin to ascend from the surface, in any convenient way, the covering glasses having been first dropped gently and squarely upon its surface so that they float, the material side being down, or in contact with the fluid. As soon as the vapor begins to rise the glasses may be removed, or they may remain longer as one pleases. The glass is then washed off in acidulated alcohol, (3 per ct. Hcl to 100 aq. dest.) until the color, red or blue, has almost disappeared from the material Wash it next in aq. dest. The tubercle bacilli retain their color, all others that may be present lose it.

This peculiarity may be made more evident by re-staining the specimen with some contrast color. For blue, an aques solution of Bismark brown; for red a solution of methyl blue or green. No heat is required this time. The glass is simply allowed to come in contact with such a solution, material side down, for a minute or so. It is then washed in absolute alcohol, and dried on a piece of white filtering paper. It can now be examined in oil, or mounted in Canada balsam. The latter must never be diluted with chloroform, but with turpentine, and oil of cloves must not be used in connection with analine colors, cedar oil being preferable. Chlorcform and oil of cloves soon reduce the color of analine dyes.

The tubercle bacilli retain the original color while all others or any other material take the secondary. Sections of suspected tuberculosis or phthisical tissues must remain in the above mentioned coloring material, which should always be freshly prepared, for 24 hours, or subjected to a moderate heat, (40 deg. C.) for an hour or so. They are to be decolorized the same as the coloring glasses; then washed in 60 per ct. alcohol; dried—spread out on a spatulum, with white filtering paper; contrast-colored, washed again in 60 per ct. alcohol, washed again as above; then in absolute alcohol, dried again; then in cedar oil; mount in balsam.

Another method, which I prefer because the coloring material can be prepared in quantity and kept on hand, and again because it acts more rapidly, has been published by Johne.

| | | |
|---|---|---|
| ℞ | Fuchsin, | 1.00 |
| | Of a 5 per ct. solution ac. carbolicum, | 100.00 |
| | Alcohol, 75 per ct. | 10.00 |

Filter and cork, never leave the bottle open. Covering glass specimens treated as above. Sections of tissues to be decolorized in a 25 per ct. solution of acid, sulfuricum. They color in 15 minutes without the aid of heat, which however intensifies the process and makes it more secure. Friedlander's "Microscopic Technik," has lately been published in English by the Appletons under the title: "The Microscope in Diagnosis," and is the most brief and handy book of the kind yet published.

### ETIOLOGY.

In the study of the pathology of any disease we have to give our attention not only to the nature or character of the disease itself as it appears in, or upon the animal organisms, but also to consider its cause with reference to the form they assume, and also their manner of acting, and further, if vital organisms, whether vegetable or animal it is the same as to what is their manner of life, and under what circumstances they flourish, and what are opposed to their continued existence. This study of the causation of disease in a general or special sense is the Alpha by which alone we may ever hope to attain the Omega towards which all the scientific methods in medicine are tending. Not only with reference to diseases of an infectious, contagious or malarial type, but also with regard to those which we technically term "sporadic," the hygienist, the honest practitioner, and the pathologist are all anxiously working to discover means for their prevention. It is first necessary that we obtain a clear understanding of the logico-pathological meaning of the words, infectious, contagious and malarial, with reference to disease. Altogether too much vagueness exists in the minds of practitioners, and I am sorry to say of pathologists and teachers also, as to the exact philosophical meaning of these words.

Contagious diseases are of necessity infectious, but all infectious diseases are by no means contagious. All malarial diseases are infectious, but the necessities to infection are of a peculiar character. To all forms of infection an either natural or acquired constitutional predisposition is necessary. Infectious diseases fall then into three great natural sub-divisions, viz., contagio-infectious, malarial and malarial-infectious diseases.

Contagious diseases are those in which the etiological moment—the inficiens, primarily and solely develops, so far as our present knowledge and experience goes, in the organism of one or more species of the animal kingdom, having what is called a natural predisposition thereto, and never outside of it. Such diseases, or rather some of them, may extend to other species, either by direct, accidental or experimental inoculation, while others cannot be thus transmitted.

The rinderpest, variolae, rabies, spotted typhus, scarlet, measles and above all in importance, tuberculosis are examples of this class.

Infectio-malarial diseases are the most difficult of differentiation and require all the acumen of the pathologist in order to place them where they belong.

They possess the peculiarity, that the predisposed organism must go to, or be in, a locality where the "genius epidemicus" reigns in order to acquire them, but they are not of necessity confined to such a locality, though strictly local diseases. They have this peculiarity, they attack a large number of individuals at the same time, according to various circumstances not necessary to discuss here, but the diseased individuals can act as the vehicles or transporters of the infectious principle, and convey it to, and infect other localities, but not individuals. The same is true of materials proceeding from such individuals. Examples:—cholera, yellow fever, the Texas cattle disease, anthrax and some other anthra-coid diseases.

It is my opinion that the so called "hog cholera" belongs in this group.

Malario-infectious diseases are such, by which the individual to acquire them has to be in certain infected localities, the natural conditions of which favor their development, but neither diseased individuals or any material from them act as a vehicle of conveyance either to other persons or other localities.

FEVER AND AGUE.—When we come to the study of any of these special classes of diseases, or in fact of any disease, we have to take into consideration three different varieties of etiological moments: if either one of them is wanting, the etiology is not complete and infection does not occur. These are known as the

*Internal, External and Sufficient Causes*—For instance: I. In cholera it is necessary that the organic or internal conditions (causes) of the individual be of such a nature as to predispose him to infection. II. If he comes into localities—external causes favorable to the support and development of the specific element—sufficient cause, by which alone the disease is produced.

We have now to make the endeavor of considering the etiology of tuberculosis from these three standpoints, but for convenience will reverse the order and discuss the external, then the internal causes, and finally the sufficient cause.

*External Causes.*—Under this head comes everything by which the individual is surrounded or which he assumes to himself—food, air, water—which is liable to exert a weakening condition upon the organism. One of the most essential of these is occupation.

In the following I can only attempt to give the very briefest testimony, and must leave it to your own consideration to fill up the various gaps which must of necessity be left in the argument. For evident reasons the subject of cohabitation must be treated in connection with the sufficient cause. Tuberculosis deserves not only to be classed as a cosmopolitan disease, but almost as a universal one, if we limit that definition to the possibility of infection common to the mammalia, which even extends to the feathered tribe.

No climate, land or people is free from this terrible destroyer, which is the cause of the death of at least 2-7 of all the people of whom we have any statistical knowledge. It would seem as if it were as old as man, entering the world, so far as history is concerned, with our species; it has followed it in all its migrations. Its origin is buried behind the mysterious veil that shades the hidden and silent records of prehistoric ages from our view. The cholera is as the breathings of an angel in comparison to it.

To us, in Massachusetts, it may seem strange to read that the extreme northern portion of Europe is notedly exempt from tuberculosis in comparison to the more central, or even southern portions. England enjoys a most unenviable reputation in this direction. In Asia, the high plateaus correspond to the general rule elsewhere, while in the valleys the disease prevails to a greater extent. The moist, hot climate of the lowlands and deltas are especially unfavorable. Europeans having the least phthisical taint soon fall victims to the disease under such conditions. It prevails to an alarming extent in China, though Hong Kong occupies an exceptionally favorable position in this regard. In the South Sea Islands, Australia and New Zealand the disease is not only frequent but very fatal among the inhabitants. As with these people, so with all other aboriginals tuberculosis has increased among them ever since, and in direct ratio to, their intercourse with Europeans. As has been said, "we send them Bibles and missionaries in the cabins of our vessels and disease and perdition in their holds." In the Mauritius and Isle de Bourbon it again prevails, while St. Helena, the Cape de Verds and Azores bear rather an enviable reputation. Livingston reports it as "very rare in Central Africa," while along the Gold Coast its only rival is dysentery. Algiers was at one time praised very highly for the favorable conditions it offered, but experience has shown that the reputation was entirely undeserved. The river bottoms and seacoast of Egypt tend to increase the bad reputation of such localities, while the dry lands of the interior are less afflicted. Those Arabs and Bedouins who retire to houses and villages for a part of the year are much more affected than those that universally live in the open country and tents. On the American continent it prevails to about the same extent among the European inhabitants, while the general rule holds true as to the unfortunate natives. In New England, Maine and New Hampshire seem to present a more favorable outlook than Massachusetts. According to Shattuck, who wrote some thirty years since, the percentage of consumption to the whole number of deaths from 1811–1820 was 4.8 per cent, while in 1840 it was three per cent.

The following figures have been kindly furnished me by Dr. Abbott, of the State Board of Health. The ratio per 1000 of the living population:

| | | |
|---|---|---|
| 1857—3.95 | 1866—2.50 | 1875—3.47 |
| 1858—3.84 | 1867—2.14 | 1876—3.23 |
| 1859—3.88 | 1868—3.22 | 1877—3.29 |
| 1860—3.70 | 1869—3.29 | 1878—3.02 |
| 1861—3.67 | 1870—3.43 | 1879—3.04 |
| 1862—3.42 | 1871—3.39 | 1880—3.08 |
| 1863—3.72 | 1872—3.62 | 1881—3.15 |
| 1864—3.78 | 1873—3.54 | 1882—3.01 |
| 1865—3.67 | 1874—3.28 | 1883—2.99 |

Which gives a death an average rate for 27 years from phthisis of 3.43 per cent. to the thousand persons.

The ratio of deaths from phthisis to other diseases has been:

| 1874. | 1875. | 1876. | 1877. | 1878. | 1879. | 1880. | 1881. | 1882. | 1883. |
|---|---|---|---|---|---|---|---|---|---|
| 16.57 | 16.44 | 16.05 | 17.41 | 15.72 | 15.79 | 15.56 | 16.14 | 15.94 | 15.71 |

For ten years, 16.13 of the total deaths, or about one-sixth. It has long been known that the climate of Florida exerted a powerful influence in some cases of phthisis, while the Gulf coast bears an equally unenviable reputation until we arrive at Texas; as we follow the coast along towards Central and South America it again becomes unfavorable. Wisconsin and Minnesota, and the high plateau of the Western States and Territories also present favorable localities in reference to this pest.

*Temperature.*—Hirsch says: That a medium temperature exerts no influence upon the frequency or extension of phthisis; the high temperature of tropical regions exerts an exactly contrary effect; this fact is not, however, so much dependent upon the excessive temperature of those regions, as upon the moisture and weaking influence of life in the tropics.

It is singular that many localities that are noted for the sudden changes of temperature that occur, are also unfavorable for the development of phthisis.

In this regard, it has been remarked that "the climate of those broad and elevated table lands which skirt the base of the Rocky Mountains is especially beneficial to individuals suffering from pulmonary diseases." The extreme northern portion of Peru, though excessively hot, is noted equally for the dryness of its atmosphere, and almost total exemption from phthisis among the inhabitants compared with the remainder of the coast.

Fourcault, Lauré and other observers have remarked that the extension of pulmonary phthisis in a people bears direct relation to the maximum humidity of the atmosphere.

Temperature of itself exerts no material influence upon phthisis.—Coolidge.

Telluric conditions exert a most important influence upon the course and frequency of these diseases; *i. e.*, the dampness of the earth is equally, if not more unfavorable than that of the atmosphere which it helps to support. The configuration of a country is what should be studied as has been already indicated.

*Hygienic Influences.*—It is almost too well known and authenticated a fact that the density of the population in a given territory exerts a most painful influence upon the extent and fatality in phthisis. The packed population of the poorer parts of our cities and manufacturing towns offer far more favorable conditions for the action of the contagio-infectious principle of tuberculosis than the sparsely populated country districts, where these elements are, as it were, diluted and dispersed by the more free-moving atmosphere.

The greater degree of cleanliness, the less concentrated and accumulated condition of the drainage in country districts, as well as the better moral and physical conditions of the people, must not be lost sight of in the consideration of this question. Poverty, luxury and vice are a trinity highly favorable to the action of bacillus Kochii. Again, the employment of persons in cities and manufacturing towns, by which people are confined in close, warm and dusty compartments, the too frequent want of exercise in the open air, are all of a maliferous character. Grinders' pneumonia and phthisis have become proverbial for such occupations. While the deaths in the State of Massachusetts for a term of years was but 2.09 per cent. for the whole population; for the city of Lowell they were 3.08 per cent. Strong, clean, healthful food is one of the essential preventives of phthisis among the poorer classes.

It is the duty of the State to guarantee this to them, as well as to so regulate the hours of labor, that these people may have time for recreation in the open air. It has been noticed also that phthisis was less frequent among mill operatives that lived at a distance and had their daily walks to and from their labors, than in the same number living in close proximity to the mills. Phthisis has always been noticed to increase with the increase of the population. Dr. Rush, a name distinguished in the early days of American medicine, wrote: "that it was scarcely known among our citizens who lived in the first stages of civilized life and who were known as first settlers. It is less common in our villages than in our cities, and increases in both with intemperance and sedentary habits."

Its frequency in jails and houses of correction has already given occasion to much thought by hygienists and philanthropists. Such conditions, while they do not and cannot, of themselves, produce phthisis, lead to the production of, or support, constitutional weakness and irritability of the respiratory tract, which prepare the ground for the specific inficiens of tuberculosis, or for caseous destructive pneumonias.

*Bovine Tuberculosis.*—Among the external causes, and certainly among the most important, is the food we eat and milk we drink, especially when the latter is the sole nourishment which we give to so many babes.

The only direct vehicle of contagious infection in this direction is to be sought in bovine tuberculosis, almost the only disease of cattle which is transmissible to man in this way. Bovine tuberculosis offers us again equally, if not stronger evidence of the value of heredity, and especially contagium, than the disease in man.

The etiological unity of tuberculosis, whenever met with in an animal organism, has been proved beyond all question. Up to within a short time tuberculosis of the milch cow engaged nearly all our attention, but since Koch has shown that the lymph cells are one of the chief vehicles by which the bacilli are conveyed over the organism, and especially since their presence has been demonstrated in the circulating blood of living individuals, the flesh of all animals in which tuberculosis is found, even in a minor degree, assumes a still graver aspect, and animal tuberculosis dare not be neglected by physicians, hygienists or legislators a moment longer. The conditions which we know as indicative of bovine tuberculosis were undoubtedly appreciated by many early observers, especially Jewish, even though they had no idea of the infectious nature of the process. Columella, 42 A. D., certainly described them. These early writers on animal diseases speak of them as nodes, ulcers and sclerotic conditions in the lungs. Chabert, Huzard and other French authors describe their macroscopic appearances very well, but had no idea of their tuberculous nature. Dupuy, 1817, was the first veterinary observer to describe tubercles in any modern anatomical sense in the lungs of animals. He is especially noted for the energy with which he advocated the tuberculous nature of equine glanders. Gurlt, 1831, considered the bovine to be equivalent to tuberculosis in man, from an anatomical point of view. He followed the Laennec school and looked upon all caseous nodules as of a tuberculous nature. Spinola defended similar views. Herring made an endeavor to differentiate between caseation and tuberculosis. He placed especially stress upon the prevalence of the latter in milch cows. Gluge was among the first to give bovine tuberculosis any very particular and critical study. He reported the disease as complicating all organs, but especially the serous membranes and lungs. Forster endorsed these views, but had no idea of the identity of tuberculosis in man and animals. Virchow, 1855, entered more particularly into anatomical study of these conditions than any of his predecessors. He adopted the word "PERLSUCHT" for the disease as an entity, which had been previously used to distinguish its appearance upon serous membranes only. He acknowledged the many resemblances between the bovine tubercle and that in man, but denied their anatomical identity, considering the former to be a lympho-sarcomatous production. From the anatomical standpoint he still adheres to this opinion, whatever he may think of the etiological entity established beyond all question, by feeding experiments, but more especially by the researches of Koch.

The first detailed description of bovine tuberculosis was given by Graumann, 1680. It is singular that this should fall in the same year that Sylvius described the macroscopical appearance of the tubercle in man. Graumann's work is decidedly clinical in its character. He looked upon it as of a syphilitic nature. It was then known, and for a long time subsquently as "morbus gallicus" or the "French disease," "Französen Krankeit." The animals were frequently described as "dicke-fat, and magere-lean—Französen." Syphilis was also honored with the same name in Germany at this time. Rühling, 1774 speaks of the endemic prevalence of the "French disease in cattle," and in 1796 said it "*w is of an hereditary nature.*"

Decrees were issued by governments against the sale and consumption of the flesh of cattle having the "French disease" on account of its supposed connection with syphilis.

Helmont asserted that sodomy was its cause in cattle; for, if criminal cohabitation could cause a venereal disease in human beings, he assumed that the criminal abuse of these functions could also produce it in cows. Kersting, 1726, spoke of its peculiar hereditary character.

In 1785, the decrees against the sale and consumption of the flesh of "French cattle" were abolished by law in Prussia, and soon after in the other German States. Frenzel, 1779, looked upon the disease as contagious and hereditary, and when fully developed similar to syphilis. "The exacerbation in the sexual organs—nymphomania—causes disturbances in the same by which a materia-peccans is produced, which is taken up by the whole system."

Virchow's anatomical views with regard to the bovine tubercle have never gained universal acceptence among veterinarians. Spinola and Haubner, but more particularly Gerlach opposed them bitterly. The latter would not accept the results of micro-histological analysis as conclusive in any sense of the term, but put more reliance upon clinical and experimental experiences. He placed great stress upon the value of hereditary influences, and was the first to prove the contagio-infectious character of the milk from tuberculous cows.

*The extension of Tuberculosis among cattle.*—I regret to say that I am utterly unable to place before you any statistics whatever as to prevalence of this disease in American cattle. Neither the Agricultural Department at Washington, nor that of any single State has ever published any such. They are not to blame. If our legislators have not intelligence enough to draft a suitable code of veterinary police and public health laws, and to organize a powerful and trustworthy veterinary police force, then the people (whom they should, but do not represent), have no right to complain of the inefficiency of those appointed to execute—nothing; and who are not supplied with sufficient funds to do anything if they would. That State which first makes an intelligent move in this direction, and thus demonstrates what *can* be done, will be a blessing to the whole Union. I venture to assert that the monetary loss to the cattle interests of this country from tuberculosis is greater than from all the other cattle diseases put together. From the general tone of conversation among my colleagues and breeders, I am led to think that it prevails more in Jerseys than in any other pure breeds, or among grade cattle. In Germany, Jerseys are rare, yet we shall see that tuberculosis is the chief disease afflicting cattle in that country.

What we desire to know is:

1st. To what extent does tuberculosis prevail in our cattle, and the exact proportion in each State?

2nd. Is there any evidence that climatic, telluric and geographical conditions exert the same influences upon bovine tuberculosis that they do on human?

3rd. Does any particular pure breed seem to have a natural predisposition to it; and if so, which?

4th. Does grading or out-crossing lessen this tendency?

5th. What is its proportion between pure breeds, and so-called "natives?"

6th. What evidence have we of its extension by the cohabitation of a tuberculosis animal among healthy ones in the same stable, contagium?

7th. What are the evidences which point to the predisposing influences exerted by heredity?

8th. Are these tendencies transmitted more by the bull than the cow, or vice-versa?

9th. What evidence can we gain that the milk is infectious to healthy calves, or young pigs?

10th. What are the losses that are caused by it?

11th. What amount of milk is sold from such cows in the State?

The only evidence that I can give of American origin is that of a Dr. Crundall, of Central New York. *He says: "the disease most prevalent among cattle in this district is tuberculosis; I have no hesitation in saying that fully 30 per ct. (?) of the grade cattle in the counties of Seneca and Ontario are affected with tuberculosis. In pure bred the percentage used to be higher, but on account of the losses breeders suffered from it, the herds that used to be kept here have been broken up, so I cannot tell much about that now."

In Germany there is a very exact veterinary police system under control of the governments exclusively, and a great many large towns and cities have public abattoirs, and others are building them, all being under veterinary supervision and the property of the municipalities. Up to the present time Bavaria has, however, been the only German State to attempt to gather any exact statistics with reference to the extension of tuberculosis in cattle. I have collected such, so far as was then possible, in my book on the "Relation of Animal Diseases to the Public Health." In 1877 the proportion of tuberculosis in Bavarian cattle was 1.62 per cent. to the 1,000. Of these 64 were 1 year old or under, 1.31 per cent.; 528 were 1 to 3 years old, 10.81 per cent.; 1,846 were 3 to 6 years old, 37.80 per cent.; 2,445 over 6 years old, 50.07 per cent. From 1877–1878 the percentage of tuberculosis to the 1,000 was 1 54 per cent.; from January 1 to December 31, 1874, 11,311 cattle (including calves) were slaughtered at Augsburg; of these 1.18 per cent. were tuberculous. In 1876, 13,241 cattle and 25,909 calves were slaughtered; 250 tuberculous. Of 231 oxen and 5,290 calves inspected at Munich before the building of the abattoir, tuberculosis was found in 235. Of 213 cattle condemned as unfit for food in Berlin, 1878, 49 were on account of general tuberculosis.

Augsburg, 1883: Whole number slaughtered, 11,829; tuberculosis, 3.12 per cent.

The first annual report of the Berlin abattoir was published in 1884. By this we find that the institution is in charge of a

* Journal Comp. Med. Vol. 5, p. 330.

Chief Veterinary Inspector, assisted by ten Chief Sub-Inspectors; an Inspector's Recorder, four Branders, a supervising Microscopic expert, a Veterinarian, four Section Veterinary Inspectors, eighty-seven Sub-Section Inspectors, and thirty persons engaged in collecting specimens for microscopic examination for trichinæ. The institution has a special microscopic laboratory, well supplied with instruments and everything necessary to the work. Whole number of animals slaughtered: Cattle, 93,837; calves, 78,220; sheep, 171,077; swine, 244,343. Number of butchers slaughtering there, 567. Number of animals by which the whole carcass was condemned on account of tuberculosis, 182 cattle; hog cholera, 72 swine; icterus, 38; dropsy, 18; general bad flesh, 9; being poorly bled, 3; ecchinococci, 1; measles, 1,621 swine; trichinæ, 216 swine; calcarious deposits, 19; actinomycosis, 15 swine.

Single organs were condemned as follows: From cattle, 21,229; calves, 816; sheep, 4,806; swine, 7,401.

Tuberculosis was found in single organs of animals, the whole of which was not condemned: Cattle, 2,613 times; calves, 2 times; swine 1,313 times.

One has but to consider the lessons which such an organized and circumspect inspection teaches, and to compare it with what is done in this country, in order to see how lamentably our governments are false to their public duties.

From it we should learn: 1. That we have in reality no meat inspection in the United States. 2. That we do not really know what diseases affect our animals, or to what extent in relation to human food.

*Causa Interna.*—Under this heading we include all those influences, whether heredity or acquired, that produce either constitutional or organic weakness, which give to any organism a predisposition to disease, *i. e.*, that produces what we technically term a "*locus minoris resistentiæ*," or point of least resistance. The subject is so vast, so delicate, and so important, that it is worthy of an especial essay, but at this time I can only touch upon certain points. In the consideration of the external cause of tuberculosis we have also considered, very briefly, the majority of influences that lead to an "acquired disposition," therefore we can now limit ourselves to those of an *heredity nature.*

We must bear in mind that it is not necessary that parents themselves be actually diseased in their lifetime; they may only possess a constitutional weakness; but they may transmit it to children, and if both parents possess it, the predisposing tendency is increased in the children, and they are very liable to anticipate their parents in the journey to that "bourn from which no traveler returns."

With reference to phthisis, it can be asserted that everything which tends to produce an irritable condition or weakness of the respiratory tract is to be looked upon as exerting a causal effect in producing that disease. "No bacillus, no tuberculosis," can be considered an axiom. Another is: No internal causes, no constitutional weakness, no tuberculosis, with due regard to the rational avoidance of contagium per contact. Without the pre-existence of these conditions, an infectious or contagious disease is shut off to the furthest limits of possibility. The biological study of any form of patho-bacterial life sufficiently demonstrates that if the cultivating medium, internal conditions is, not suitably composed chemically, if it has not the right degree of moisture, if the temperature offered is not conformable to their biological necessities, the bacteria cannot thrive; they die out. This is as true of the tubercle bacillus as any other. In fact they possess what we may call more constitutional delicacy, perhaps are more dainty, in these directions, than almost any form that we know of. A vigorous, healthy, respiratory tract does not offer these conditions. A weak one does, or, upon very slight irritation, may.

The existence of this last fact is the curse of our species. Through ignorance, through heedlessness, through the sensuous attractions of a pretty face, a sylph-like form; the human race is, and has been, doing its best to make true and everlasting the saying of the biblical writer, that "the sins of the father shall be visited on the children." Can the "curse" be rooted out? Yes, in time, by the combined action of the intelligence of the land. To do this one thing is necessary above all. Man must know himself. He must know that he is a subject to the material laws of his being as every other created thing. Once, the religious-philosophical discussions were full of what was and what was not "free will?"

My idea of "free will" is the ability to study the phenomena of nature, including animal life, which the mind of man has acquired in its long evolutional development. Having studied these phenomena, we have gradually acquired the ability to read the laws upon which their appearance depends. We have found that every created thing, animate or inanimate, is bound with chains of iron, scientifically speaking, by its structural, constitutional or physical direction to those laws. Even the highest representatives of animal intelligence cannot depart from them. We call this property "instinctive action." Man of to-day is the only exception. He has acquired a knowledge of these laws, even as regards himself. He can subject himself to them, or not, as he wills. This is alone his physiological superiority over the lower animals. It is all the "free will" there is. Apply the principle to humanity in relation to phthisis. In Europe the governments forbid the use of animals having constitutional weakness for breeding the higher grades. American and English breeders are gradually learning, from disastrous experience, that individual cows, or bulls, or even families do not pay to be used for breeding, on account of tuberculous tendencies. Does it pay to breed these tendencies in our children? Should not the State represent the acme of intelligence and control and forbid it by law? The influence of heredity in producing constitutional weaknesses predisposing to phthisis has long been known to the medical profession. It is time that we began to concentrate our energies and in unison with the State make an endeavor to gather well-selected statistics in this regard.

Tuberculosis having been established to be a unit, it may be well to see what observations the veterinary profession has made with regard to heredity, a few of which have already been noticed. Krunitz, 1778, wrote that: "After suffering great losses for years in consequence of the use of tuberculous animals for breeding purposes, various owners have concluded to exclude all such animals from such uses." Lyditin says: "In order to prove that the disease can extend directly from tuberculous parents to offspring we must be able to show that evidence of the same exist in the young at time of birth, or during intra-uterine life, which would lead us to infer that either the ovum or the fœtus had been affected ante-partum.

Heredity is not without influence upon the extension of phthisis. The predisposition can be given to the young by father, mother or both. Transmission of the inficiens to the ovum or fœtus, either causes sterility or abortion."

Johne has demonstrated for the first time, the presence of the specific bacilli in the lungs of a calf aborted in the eighth month of pregnancy. Adam says that, "although tuberculosis seldom develops in the fœtus, it has been sufficiently proved that, a tuberculous cow can transmit the tendency to this disease to its offspring." Busch, 1880, demonstrated the lungs of a sucking calf full of tubercles; 1878, 1 such was found at Augsburg; 1880, 5 were found at Nürnburg. Virchow, reports a case of tuberculosis of the ovaries and tubes of a sucking calf. Semmer, notices 5 cases of pulmonary tuberculosis in the fœtus of cows; they were all aborted; one at three months, another at six, another at eight; while the others were but just dropped. He says: "These cases prove to me, that tubercles can develop in the fœtus during pregnancy." Jessen found the lungs of a three months' old calf full of tubercles.

The fact already alluded to, of the intra-vital presence of tubercle bacilli in the blood, and furthermore the fact, that other forms not so minute have been shown to pass from mother to fœtus explain this dreadful phenomena. It is now left to demonstrate the bacilli in the blood of the mother and in that of the aborted fœtus at the time of the accident.

### Causa Sufficiens.

*Contagium.*—Having endeavored to draw your attention to the causes or conditions which exert the chief influence in preparing the organism to become the seat of phthisical processes, and without which there can be no phthisis, it now remains for us to consider the essential and direct cause of tuberculosis phthisis; *i. e.* its sufficient or exciting cause.

You must not forget, however, what I have so many times placed stress upon, that, under ordinary circumstances, no predisposing conditions, no tuberculosis. Hence I say to you that, notwithstanding the immense importance of the study of mycology with reference to disease; notwithstanding the fact, that, our respective governments must be branded as traitors to their highest responsibilities, viz., the public health, for not establishing laboratories in connection with boards of health in order that researches may be made into the cause and nature of disease, still you as practical physicians, and my colleagues, as pathologists, must not be false to the terrible responsibilities which we assumed toward the public when we entered the medical profession. We must never lose sight of the value of those external and more especially the internal conditions which find their chief support in the heedlessness with which the responsibilities of parentage are assumed, entirely regardless of the welfare of its product, our babes.

Ziegler, one of the most recent authorities, says that tuberculosis is an infectious disease, which is anatomically characterized by the development of noduli. This definition is not correct in the light of the latest research. Glanders has every one of those characteristics, and one more, which is also shared by tuberculosis. The presence of bacilli in the nodulus. For us tuberculosis is a contagious disease, characterized by the development of noduli, which extend, apparently, per continui-tatem to adjoining tissues, which are of cellular character, including giant cells, having a peculiar nuclear arrangement, which finally undergo cheesy degeneration, which leads to their destruction, and finally, and above all, which owe their origin to certain bacilli, having a peculiar and specific reaction to certain specific methods of coloring never found possessed by other bacteria in the same tissues, and under the same circumstances.

The idea that tuberculosis *could be* transmitted to other persons or animals, is much older than that it is a contagious disease per se. Klenke, 1843, was the first to demonstrate that it could be transmitted to rabbits, by inoculating them with material from tuberculous cadavers. These experiments passed into forgetfulness until Villemin again repeated them, 1865, and caused the question to be one of all exciting and vital importance. It soon became shrouded in ambiguity, from the fact that an immense number of observers found that noduli not only existed in animals, but also could be produced by all manner of indifferent materials, either introduced under the skin into veins, or the great cavities of the body. Among them may be mentioned glass, setons, pus bonum, glanders material, dust, healthy and freshly slaughtered flesh, in fact almost everything that could be thought of. In rabbits especially, tubercles developed almost every time.

The experiments of Herard and Cornil, are especially interesting because, they serve to illustrate a point which I have raised against the teachings of the bacteria-school. They used substances derived from cases of caseous-broncho-pneumonia in man.

They were followed by such negative results as to lead them to deny its identity with tubercular phthisis. They again repeated them, but with material from another person. The results were positive and they changed their opinion. The fact is that, in the first case the person was not tuberculous, the bacteria had not entered into the material. The ground was ready waiting for infection. In the second case, and in all where positive results follow, this had occurred.

The material used, and the material produced must be examined, before in the first case, and post in the second in order to prove this question.

Marcet produced tuberculosis with the blood from a phthisical patient. Klebs went over the whole field and concluded that, "the noduli which develop on the inoculation of indifferent substances could not be true tubercles, since they did not possess the character of progressive infection." By this he means, extension per continuitatem, and not disseminated tuberculosis over all the organs of the body, as some have objected. Cohnheim and Frankel made very extensive experiments at the pathological laboratory, Berlin, with indifferent materials. Results positive. "The noduli produced had all the characteristics of true tubercle; hence tuberculosis is not a specific disease."

They repeated their experiments at Breslau, under far more favorable circumstances than those offered by the germ-infected laboratory at Berlin. They used the anterior ventricle of the eye of albino rabbits, and for the first time, thus supplying us with a most favorable "locus operationis." Results positive, ergo, tuberculosis is a specific infectious disease.

Haensel showed, that, tubercle followed inoculation made with syphilitic material. Buhl inaugurated another theory of infection. As he almost invariably found caseous centres which ante-dated the eruption of tubercles in phthisical patients, he concluded that, during, or by, the processes of cheesy metamorphosis, a "materia peccans" was produced, which being absorbed, acted as a specific irritant, to predisposed tissues, which resulted in the development of tubercles.

The identity of scrofulosis and tuberculosis again found advocates on this account. Spina sums up the results of this period of inoculative experiment as follows: "In view of the fact then, that not one case is reported in which healthy animals acquired tuberculosis by being confined with sickly ones (?) (he had not looked over verterinary literature), also, of the fact tubercles are, as a rule, found in close proximity to the 'locus inoculations;' and in view of the fact that, particles of the inoculated materials have been found in the tubercles produced, we are justified in concluding, that the specific character and contagiousness of tuberculosis has not been proven."

The first to really prove beyond all question of doubt, that tuberculosis did produce, or contain, an infectious principle, was a veterinarian. Gerlach's experiments were made with the milk from tuberculous cows, and not with phthisical material taken from dead persons. He says: "Having a cow afflicted with tuberculosis, it was resolved to test the question, whether the milk from such a cow is capable of producing a similar disease in young animals when fed upon it?" The results of these experiments on quite a number of young animals were partly positive, and in part negative; but they proved that the milk from such cows can produce tuberculosis in young animals when fed upon it. Bollinger (Zeimssen's hand-book, 1876), sums up the results of the milk-feeding experiments up to that time: 3 pigs, 1 positive; 2 doubtful. 3 calves, 2 positive; 1 died prematurely. 1 lamb, 1 positive. 2 dogs and 2 cats, negative. 14 rabbits, 2 positive; 6 negative. The milk given to the 6 in which negative results followed had been previously boiled.

Albert reports a case of practical observation, in which a far mers wife fed a litter of pigs on milk from a tuberculous cow that was so bad she did not dare u-e it. The pigs died of tuberculosis, as revealed by a microscopical examination. *Bang however, gives the most conclusive proof of the infectiousness of the milk from tuberculosis cows, and shows why such experi-

*Journal Comp. Med. vol. 6, p. 143.

ments have occasionally failed. In every one of his cases the tuberculosis processes had extended to the udder; "mammitis tuberculosa;" and in every one of them he was able to demonstrate the presence of the specific bacilli in the milk before it was given to the calves. Results all positive. Johne has shown that it is always possible to differentiate between simple (garget) and tuberculous mammitis in the cow by the examination of the milk. I can confirm that testimony! Nocard found the specific bacilli in the milk of 11 cows with mammitis tuberculosa.

Johne reports 322 feeding experiments made with all sorts of tuberculous material; result 43.5 per. ct. positive; 51.1 per ct. negative.

These experiments were divided as follows:—

117 Animals fed with tuberculous material from calves, 61.5, posible; 34.2 negative.

46 animals fed with cooked meat from a tuberculous cow, 13.1 possible; 86.9 negative.

91 animals fed with milk, " " " 30.7 positive; 59.3 negative.

1 animal fed with milk from a tuberculous rabbit, 100.0 positive.

25 animals fed with tuberculous material from man, 30.0 positive; 64.0 negative.

33 animals fed with the tuberculous " " hogs, 53.0 positive: 47.0 negative.

2 animals fed with the tuberculous " " rabbits, 50.0 positive; 50.0 negative.

2 animals fed with the tuberculous " " monkeys, 100.0 positive.

5 animals fed from the tuberculous " " hens, 100.0 positive.

Touisaint, Chauvau and others all bear testimony that, the continuous feeding of milk from a tuberculous cow to swine will cause the disease. I have fed 3 hens with tuberculous sputum from man, they are already dead—cause tuberculosis.

Johne reports that, a flock of ten hens were daily fed with crusts of bread and leavings from a man having phthisis, the hens died of tuberculosis one after another. Zschokke relates of "a pet cat, that constantly accompanied and slept in the bed with a phthisical old maid. The cat died of tuberculosis." Ogston (British Med. Journal, 1884), reports a case which is full of warning. "In an entirely healthy family of ten persons without a discoverable trace of hereditary predisposition. One son went away from home and became phthisical. He returned home to die. The two sisters who took care of him, and a brother who slept with him, all followed him to the grave in a short time from tubercular phthisis." *Reich relates another case pregnant with warning and instruction. "From July 11th, 1875, to Sept. 29th, 1876, ten children died of tubercular meningitis, that were born between April 4th, 1875, and May 10th, 1876. These ten children were brought into the world and taken care of by a midwife, Frau Sanger, who had a habit of blowing into the mouth of all recently born babes. There was no ascertainable disposition to tuberculosis in any of the ten children. In the practice of another midwife in the same village, not one single child died or sickened of tubercular meningitis in the same time. The nurse Sanger had tubercular phthisis. In three of the children that died of tubercular meningitis, that came into my care, the disease began with bronchitis. Meningitis tuberculosa is not endemic here, only two cases having occurred from 1866 to 1874."

German veterinarians have given many cases of tuberculosis extending over the cattle of a stable from one diseased animal. Albrecht tells of a farm on which "the owner kept 14 milch cows, a bull and 4 young animals. The stables were always kept full The animals were under his observation for 3 years, during which time tuberculosis was always present. He traced it to 2 old cows that had been there the whole time. New ones introduced were healthy when bought." He gives still another case where the control was more exact. In this 19 animals died of tuberculosis in 10 years. The cause of the disease was a calf that had had a tuberculous mother. It was in the stable from 1864 to 1869, but never thrived well. In '69 it was killed and found tuberculous. In '69 other cows began to cough, and the disease gradually extended among them as was proved by necroscopi-

* Berliner Klin. Wochenschrift. 1878.

cal examinations. Lydtin's works are replete with such evidence.

I will close this testimony with one more case of the kind in man. *Ollivier reports two cases of undoubted contagious infection in children, that had been previously healthy and were free from all taints of hereditary predisposition. The infection was due to living in the same room with phthisical children, and breathing the same air.

We have seen up to 1884 there were still some doubts as to the specific contagiousness of tuberculosis, and that experimental pathology had been unable to decide the question beyond all doubt. This was left to Robert Koch to do. He discovered in the sputa and tissues of tuberculous persons, and animals (cattle), bacterial organisms that were invariably present, and which had as shown, a peculiar and specific reaction to certain methods of coloring. He isolated these bacteria and cultivated them in a pure form. He was enabled to produce tuberculosis with them at will, in animals, by subcutaneous inoculation, aspiration, and by putting them in their food. He carried these cultivations through many generations, and was enabled to produce tuberculosis with them. In every case he proved the bacilli to be present in the diseased tissues of the animals experimented upon. He used this material as a means of inoculation itself; he also again made cultivations from them, and again produced tubercles in other animals. He therefore demonstrated, tuberculosis to be a specific infectio-contagious disease, due to the bacillus which bears his name.

These bacilli have been found in the urine and fœcal discharges of persons having tuberculosis of the appropriate organs, as well as the sputum. They have also been found in the catarrhal discharge of the uterus in a cow, and were afterwards demonstrated in that organ.

Koch describes the tubercle bacilli, "*as always* appearing as rods, which the name indicates; having a length of from 1–4th to 1–2 the diameter of a red blood cell. As inconstant as they are in length, even so constant are they in their transverse diameter. They are not however completely straight rods, but frequently have a notched or very slightly curved appearance,

*L'Union Medicale, No. 70, '85.

which sometimes indicates an inclination to the beginning of a spiral form. These variations from a direct line are of some value in differentiating between tubercle and other similar rod bacteria. They develop spores, which sometimes take on a streptoid form.

### PROPHYLAXIS.

What should be done in order to prevent the further extension of tubercular phthisis among the people has already been sufficiently indicated, hence, all that remains for me to do is to outline to you how it must be done. In order to prevent a given disease it is necessary that we take into the most minute consideration everything that can possibly have the most remote connection with its etiology. This can only be expressed by the word *research.* To carry out this threefold purpose it is necessary that some one authority, representing the accumulated intelligence of the people on the subject of public health should be selected by the State.

Such an organization we call a state Board of Health. The work of such a board in the width and breadth of its responsibilities has scarcely dawned upon the minds of the people. Such boards are too often looked upon as honorary retreats in which to shelve useful or useless politicians. The only attribute that should make men candidates for such appointments should be fitness for the work. Such boards should be composed of an eminent jurist; the ablest hygienic special engineer attainable; a competent chemist; a specialist in public health as a medical man; and the best educated and qualified veterinarian in the State, upon matters of public health, and contagious animal diseases. The latter should be known as the State Veterinarian, and should be connected with the board of agriculture also. The men should be liberally salaried according to the time and work required of them.

The responsibililies are beyond those of any persons employed by the state.

The governor should be a member of the board, so that he be intimately acquainted with its workings; its wants and necessities, in order that he may intelligently recommend legislation. The legislature should be more liberal to it, than to other departments of government.

There is not a laboratory in the United States fitted up as it should be and liberally supplied with funds; under the control of competent experts, where researches into the cause and nature of disease are being made, or means of prevention sought for. Yet it is possible to discover a vaccine virus in a pure form that can be cultivated and dispensed in unlimited quantities, without having recourse to animals that may have the germs of tubercular phthisis coursing through their veins. It is possible to produce a practical virus against the contagious-lung-plague of cattle that has cost us millions, and will cost us many more; it is possible in this way to reduce the losses from hog-cholera to the lowest possible minimum; it is possible to find out the original source of trichnosis, and thus instruct our farmers how to feed and raise their hogs, so that the stigma may be raised from American pork, and much suffering and many lives saved in our own species. All these things are possible, and many more of a kindred nature, highly probable. But scarcely a dollar is devoted to it. Legislation should be made upon the recommendations of the Board of Health, and not according to the wills of an unreflecting and scarcely interested public, or upon the whims of legislators.

The State should be districted off into health sections, each of which should have its medical and chief veterinary sanitary official, and the necessary number of sub-officers. These men should be elected, and vacancies filled, on account of displayed competency only. The State Board of Health should be the appointing and examining body. Local boards in cities or towns should be appointed by the State Board and subject to it, and not by or to the local governments. All laws should be State, not local, so that their tenor and execution may be the same and contradictions or local inefficiency avoided. Every local, health officer should be paid for his time and services from the State funds, and the local authorities should hold the state Board of Health responsible for all neglects of this branch of the public service. The practice of medicine should be so regulated by the State that the people could if they desired, distinguish the accredited from the unaccredited man. The right of selection must be left to the people.

Malfeasance in practice should be regulated by the State, and the further right to practice in the State prohibited to the perpetrators thereof, whether a regular or irregular practitioner. All diseases should be scheduled, and all practitioners, regular, veterinary and irregular, be obliged to notify the respective local authorities of every suspicious case of disease of a contagious or infectious character, under penalty of the law. Statistics should be gathered not only with reference to the number and cause of death, but also of everything having etological connection with it. This is especially true with regard to tubercular phthisis.

We should know each year, how much occupation, manner of living, heredity and contagion have to do with the extension of this destroyer among every class of people.

Marriage inspection boards would be to day too ultra a proposition, but the medical profession should constitute itself into a marriage advisory board. If breeders of animals have intelligence enough to exclude from such purposes animals with tuberculous taints, it is about time we began to apply such principles to our own species, and thus endeavor to gradually lesson phthisical tendencies in it. The trouble does not lie in the intentions of the people, but in their ignorance. Boards of health should get the medical profession to co-operate with them, and have competent men lecture upon hygiene and the prevention of disease during the winter months all over the State. The people want knowledge and it is the duty of the state to supply them with the bread of life. It is the duty of the state to guarantee the people that, the animal products which they use as food are free, not only from disease itself, but disease producing elements, as well as their water supply.

Our milk inspection is a semi-farce. If the cows are tuberculous, or otherwise diseased; if they are improperly housed or fed, what a humbug it is to watch the stream from its fountain head to the consumer, and leave the spring itself entirely out of consideration.

The State should know the exact hygienic condition of every animal in it. By this means alone can it know how much it annually loses from this cause, and to what extent contagious or infectious diseases prevail. It cannot know what it should seek to prevent until it knows what exists, how it came to pass, and how it gets spread about.

The fact that the bacilli of tuberculosis have been found in the blood indicates that they must be in the flesh, hence, no part of such animals, or derivates from them, should be sold for human food; yet thousands of them are, and hundreds of quarts of milk from diseased cows dispensed over our cities, or ignorantly consumed by the people themselves.

We should know the exact condition of every animal slaughtered, both before and after death; we should know the condition of each individual organ, and as shown by the Berlin abattoir, an exact statistic should be kept of the results of such examinations. The state should compel the erection of municipal abattoirs in every city and large town, used for every so many thousand people in the country districts. All animals destined for human food, whether for owners use or not, should be slaughtered therein.

They should be all brick, with asphalt floors, iron doors and no wood about them. The chief inspector and technical assistants should be qualified veterinarians appointed by the state board of health, but paid by the local authorities. No animal fairs or markets should be allowed to be held unless a State veterinarian were present to supervise the health of the animals and their freedom from contagious disease. The inspection should be especially extended to the animals of visitors, showmen, pedlers or dealers.

Thus I have endeavored to sum up the preventive work of the noblest profession opened to intellect and handwork of man. Unless we one and all put all our power to the wheel, and aid in the fulfilment of this grandest of all works we are false to our responsibilities and traitors to our race.

### BIBLIOGRAPHY.

Hippocrates Werke; Carswell, Pathology Atlas; Schonlein, Pathologie und Therapie; Henle, Rationelle Pathologie; Rokitansky, Pathologische Anatomie; Virchow, Cellular Pathologie; Herch, Historisch-Geograpische Pathologie; Perls, Allgemeine Pathologische Anatomie; Cohnheim, Vortesungen u Allgemeine Pathologie; Klebs, Pathologische Anatomie; Birsch-Hirschfeld, Pathologische Anatomie; Ziegler, Pathologische Anatomie; Samuels, Allgemeine Pathologie; Mitthielungen, a. d., Reichs-Gesunheits Amt., Berlin; Cornil-Balus, Les Bacteries, Ziemsen's Handbuch; Lungue-Kankheiten,

Zoönosen; Fortschrift d., Medicin; Berliner Klinische Wochenschrift; Deutsche Klinische Wochenschrift; Recueil, d., Med. Veterinair; Archiv. fur Thierheilkunde, Berlin; Zeitschrift für Thiermedicin, Munich; Bovine Tuberculosis in Man, Creighton; History of Tuberculosis, Saltler; Bruchmüller, Path. Zoötomie; Virchow's Archivs.

